# *Tropaeolum majus* R2R3 MYB Transcription Factor TmPAP2 Functions as a Positive Regulator of Anthocyanin Biosynthesis

**DOI:** 10.3390/ijms232012395

**Published:** 2022-10-17

**Authors:** Xiaoping Wang, Wei Wang, Siyu Chen, Yuji Lian, Shucai Wang

**Affiliations:** Laboratory of Plant Molecular Genetics & Crop Gene Editing, School of Life Sciences, Linyi University, Linyi 276000, China

**Keywords:** nasturtium, *Tropaeolum majus*, anthocyanin biosynthesis, transcription factors, TmPAP2, TmTTG1, TmGL3

## Abstract

Anthocyanins are an important group of water-soluble and non-toxic natural pigments with antioxidant and anti-inflammatory properties that can be found in flowers, vegetables, and fruits. Anthocyanin biosynthesis is regulated by several different types of transcription factors, including the WD40-repeat protein Transparent Testa Glabra 1 (TTG1), the bHLH transcription factor Transparent Testa 8 (TT8), Glabra3 (GL3), Enhancer of GL3 (EGL3), and the R2R3 MYB transcription factor Production of Anthocyanin Pigment 1 (PAP1), PAP2, MYB113, and MYB114, which are able to form MYB-bHLH-WD40 (MBW) complexes to regulate the expression of late biosynthesis genes (LBGs) in the anthocyanin biosynthesis pathway. Nasturtium (*Tropaeolum majus*) is an edible flower plant that offers many health benefits, as it contains numerous medicinally important ingredients, including anthocyanins. By a comparative examination of the possible anthocyanin biosynthesis regulator genes in nasturtium varieties with different anthocyanin contents, we found that *TmPAP2*, an R2R3 MYB transcription factor gene, is highly expressed in “Empress of India”, a nasturtium variety with high anthocyanin content, while the expression of *TmPAP2* in *Arabidopsis* led to the overproduction of anthocyanins. Protoplast transfection shows that TmPAP2 functions as a transcription activator; consistent with this finding, some of the biosynthesis genes in the general phenylpropanoid pathway and anthocyanin biosynthesis pathway were highly expressed in “Empress of India” and the *35S:TmPAP2* transgenic *Arabidopsis* plants. However, protoplast transfection indicates that TmPAP2 may not be able to form an MBW complex with TmGL3 and TmTTG1. These results suggest that TmPAP2 may function alone as a key regulator of anthocyanin biosynthesis in nasturtiums.

## 1. Introduction

Nasturtium (*Tropaeolum majus*) is an annual bushy flowering plant from the order of Brassicales, in the Tropaeolaceae family; it is native to the Andes and is widely disseminated throughout South America [1,2]. Nasturtium is one of the most popular edible plants, with all parts being edible, including the leaves, flowers, and unripe green seeds; it offers many health benefits as it contains a large amount of vitamin C and lutein [3,4]. In addition, nasturtium leaves are used in traditional medicine to treat different diseases, including asthma, urinary tract infections, and cardiovascular disorders [5,6]. Pharmacological studies have shown that the extracts from nasturtium leaves and flowers perform antithrombin, antihypertensive, and antibacterial activities [7,8,9,10]. Due to its medicinal importance, nasturtium was named the medicinal plant of the year in 2013 (https://www.live-native.com/nasturtium/, accessed on 1 September 2020). In addition, a few available toxicological studies have demonstrated that there is no acute toxicity or genotoxicity in nasturtium plant extracts [2,11].

The therapeutic and health benefits of nasturtium are believed to be related to its high levels of secondary metabolites, including glucosinolates [12,13,14], terpenoids, such as lutein and carotenoids [15], vitamin C, and phenolic compounds, such as flavonoids and anthocyanins [1,11,16].

Anthocyanins are an important group of non-toxic water-soluble natural pigments that are found in flowers, vegetables, and fruits, and that have been listed as natural colorants (code E163) by European Union legislation [17]. Several studies have shown that anthocyanins have antioxidant and anti-inflammatory properties, and may have the potential to prevent cardiovascular disease, colon cancer, and diabetes [18,19,20,21,22]. Anthocyanins are enriched in some fruits and vegetables, such as blueberries, mulberries, blackberries, and purple cabbage, and a high intake of anthocyanins can be achieved by regularly consuming these fruits and vegetables [17,19].

Anthocyanin biosynthesis is a specific branch of the phenylpropanoid biosynthesis pathway and is largely conserved in different plants [23,24,25]. Anthocyanins are synthesized from coumaroyl-CoA, whereas the conversion of phenylalanine to coumaroyl-CoA is catalyzed sequentially by several general enzymes in the phenylpropanoid biosynthesis pathway, including phenylalanine ammonia lyase (PAL) [26], cinnamic acid 4-hydroxylase (C4H) [27], and 4-coumarate CoA ligase (4CL) [28].

Enzymes encoded by anthocyanin biosynthesis genes (ABGs), including chalcone synthesis (CHS), chalcone isomerase (CHI), flavanone 3-hydroxylase (F3H), dihydroflavonol reductase (DFR), anthocyanidin synthase/leucoanthocyanidin dioxygenase (ANS/LDOX), and UDP-flavonoid glucosyl transferase (UFGT), sequentially catalyze anthocyanin biosynthesis [23,24,25]. CHS, CHI, and F3H function in the early anthocyanin biosynthesis steps, where CHS converts coumaroyl-CoA to naringenin chalcone [29]. The chalcone is isomerized into flavanones by CHI [30], and the flavanones are then converted to dihydroflavonols by F3H [31]. DFR, ANS, and UFGT function in the later anthocyanin biosynthesis steps, where DFR converts dihydroflavonols to flavan-3,4-diols (leucoanthocyanins) [30], which are then converted to anthocyanins by ANS and UFGT [32,33,34].

Several different transcription factors, including the R2R3 MYB transcription factor, bHLH transcription factor, and a WD40-repeat protein have been shown to regulate anthocyanin biosynthesis via regulating the expression of ABGs. In *Arabidopsis*, MYB11, MYB12 and MYB111, three R2R3 MYB transcription factors function redundantly to regulate the expression of the early biosynthesis genes (EBGs), including *CHS*, *CHI*, and *F3H* [35,36], whereas the expression of late biosynthesis genes (LBGs), including *DFR*, *ANS*, and *UFGT*, is regulated by the MYB-bHLH-WD40 (MBW) activation complexes formed by the WD40-repeat protein Transparent Testa Glabra 1 (TTG1), a bHLH transcription factor Transparent Testa 8 (TT8), Glabra3 (GL3), or the Enhancer of GL3 (EGL3), and the R2R3 MYB transcription factor, Production of Anthocyanin Pigment 1 (PAP1), PAP2, MYB113, or MYB114) [37,38,39,40,41,42,43]. On the other hand, the expression of both EBGs and LBGs in maize is regulated by the MBW activation complexes, indicating that the regulation of anthocyanin biosynthesis in dicots may be different from that in monocots [23]. However, recent experiments indicate that the regulation of both EBGs and LBGs by the corresponding MBW activation complexes may be conserved in plants such as *Arabidopsis*, *Ipomoea purpurea*, rice, peppers, eggplant, and *Freesia hybrida* [44,45,46,47,48].

In this study, we report the identification of TmPAP2, a nasturtium R2R2 MYB transcription factor that functions as a key regulator of anthocyanin biosynthesis. We found that *TmPAP2* is highly expressed in “Empress of India”, a nasturtium variety with high anthocyanin content, while the expression of *TmPAP2* in *Arabidopsis* promoted anthocyanin biosynthesis. The RT-PCR results show that the expression levels of EBGs and LBGs, as well as some of the biosynthesis genes in the general phenylpropanoid pathway, are increased in the *TmPAP2* transgenic *Arabidopsis* plants, as well as in the “Empress of India” nasturtium. Consistent with this finding, the protoplast transfection assays show that TmPAP2 functions as a transcription activator. However, transfection assays in the protoplasts indicate that TmPAP2 may not able to form an MBW complex with TmGL3 and TmTTG1, suggesting that TmPAP2 may function alone to regulate anthocyanin biosynthesis via activating the ABGs and biosynthesis genes in the general phenylpropanoid pathway.

## 2. Results

### 2.1. TmPAP2 Is Highly Expressed in a Nasturtium Variety with High Anthocyanin Content

**Figure 1:**
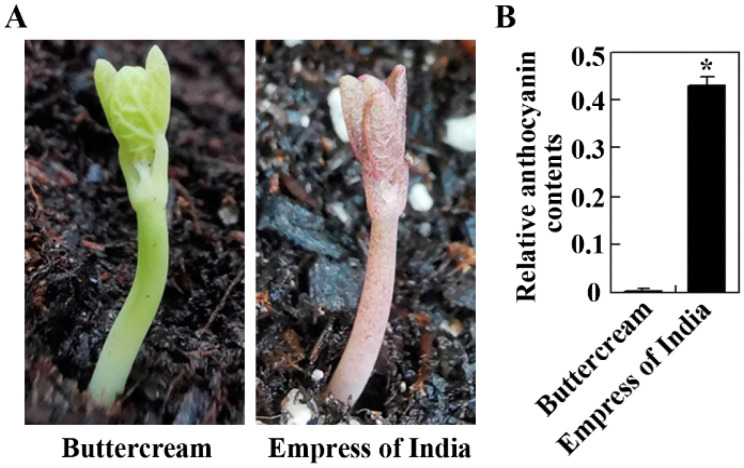
Anthocyanin accumulation in seedlings of the “Empress of India” and the “Buttercream” nasturtiums. (**A**) Seven-day-old soil-grown seedlings of the “Buttercream” and the “Empress of India” varieties. Pictures were taken using a digital camera. (**B**) Relative anthocyanin contents in the seedlings of the “Buttercream” and the “Empress of India” varieties. Data represent the mean ± standard deviation (SD) of two biological repeats. * Significantly different from the “Buttercream” nasturtium (*p* = 0.001).

Anthocyanins are one of the most medicinally important ingredients in nasturtium [1,9,11,16]. Based on the phenotypic examination of the different nasturtium varieties obtained, we found that the “Buttercream” and the “Empress of India” varieties show opposite phenotypes in terms of anthocyanin production; the “Buttercream” variety produced very little, whereas the “Empress of India” variety produced many more anthocyanins, as indicated by the color of the seedlings (Figure 1A). Quantitative analysis showed that the anthocyanin content in the leaves of the “Empress of India” variety was about 200-fold that in the “Buttercream” variety (Figure 1B).

**Figure 2:**
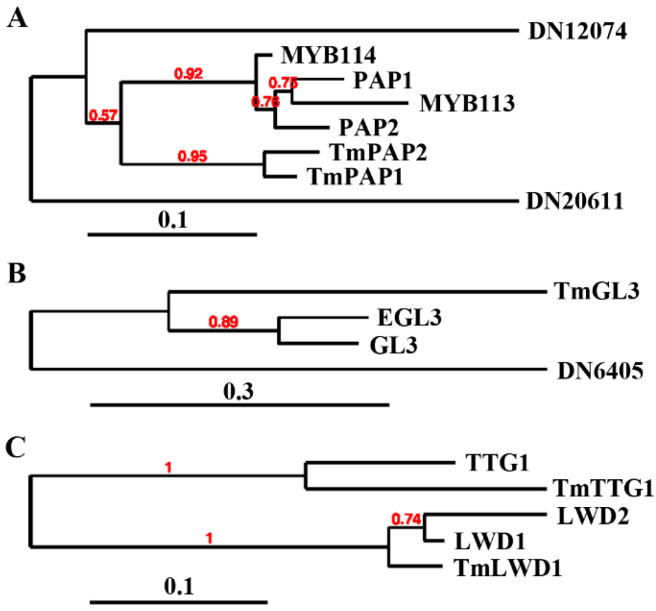
Nasturtium homologs of the *Arabidopsis* MBW complex proteins. (**A**) Phylogenetic analysis of anthocyanin biosynthesis-regulating R2R3 MYB transcription factor in *Arabidopsis* and their homologs in nasturtiums. (**B**) Phylogenetic analysis of the anthocyanin biosynthesis-regulating bHLH transcription factors in *Arabidopsis* and their homologs in nasturtium plants. (**C**) Phylogenetic analysis of the anthocyanin biosynthesis-regulating WD40 transcription factor in *Arabidopsis* and its homologs in nasturtiums. The amino acid sequences of *Arabidopsis* proteins were obtained via Phytozome (https://phytozome-next.jgi.doe.gov, accessed on 1 May 2021), while the amino acid sequences of nasturtium proteins obtained in this study are listed in File S1 in the Supplementary Materials. Phylogenetic analysis was performed on Phylogeny (http://www.phylogeny.fr/simple_phylogeny.cgi, accessed on 1 August 2022).

To identify the regulator involved in the regulation of anthocyanin biosynthesis in nasturtiums, we performed a comparative RNA-sequencing analysis using RNA isolated from the leaves of the “Buttercream” and “Empress of India” nasturtiums, and a total of four nasturtium homologs of anthocyanin biosynthesis regulators in *Arabidopsis*, including the R2R3 MYB proteins PAP1, PAP2, MYB113, and MYB114; the bHLH transcription factors GL3 and EGL3 and the WD40 repeat protein TTG1 were identified (File S1 in the Supplementary Materials). Among them, two are closely related to PAP1, PAP2, MYB 113, and MYB114; we named them TmPAP1 and TmPAP2, respectively (Figure 2A). One is closely related to GL3 and EGL3; we named it TmGL3 (Figure 2B). The other is closely related to the WD40 repeat protein TTG1; we named it TmTTG1 (Figure 2C). As LWD1 and LWD2 are WD40 proteins that are closely related to TTG1, we also identified their nasturtium homolog, i.e., TmLWD1 (Figure 2C), for phylogenetic analysis to ensure that the TmTTG1 identified in the test is the homolog of TTG1. 

**Figure 3:**
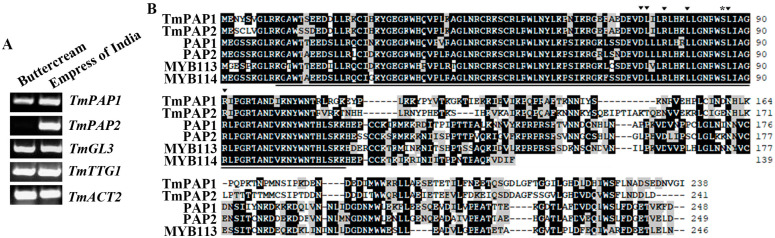
Expression of the MBW complex component genes in the “Buttercream” and the “Empress of India” nasturtium varieties and the amino acid sequence alignment of TmPAPs with *Arabidopsis* anthocyanin biosynthesis regulating R2R3 MYB proteins. (**A**) Expression of *TmPAP1*, *TmPAP2*, *TmGL3*, and *TmTTG1* in the “Buttercream” and the “Empress of India” nasturtiums. RNA was isolated from 7-day-old soil-grown seedlings, and RT-PCR was used to examine the expression of the genes. The expression of *TmACT2* was used as a control. (**B**) Amino acid alignment of TmPAPs with *Arabidopsis* PAP1, PAP2, MYB113, and MYB114. Underlines indicate the R2R3 domain, triangles indicate the amino acid required for the interaction of MYBs with bHLHs, and the star indicates the amino acid critical for DNA binding in GL1.

According to the RNA sequencing results, among the nasturtium homolog genes, both *TmPAP1* and *TmPAP2* have relatively higher expression levels in the “Empress of India” variety, whereas *TmPAP2* is undetectable in the “Buttercream”, but *TmGL3* and *TmTTG1* have similar expression levels in the “Empress of India” and the “Buttercream” (Table S1 in the Supplementary Materials). RT-PCR results further confirmed that *TmPAP1* has a relatively higher expression level in the “Empress of India” compared with the “Buttercream”, while *TmPAP2* was highly expressed in the “Empress of India” but undetectable in the “Buttercream”, whereas the expression levels of *TmGL3* and *TmTTG1* were largely similar in both the “Empress of India” and the “Buttercream” varieties (Figure 3A).

Amino acid sequence alignment showed that the R2R3 MYB domain is highly conserved in TmPAPs and the R2R3 MYB regulators of anthocyanin biosynthesis in *Arabidopsis*, including PAP1, PAP2, MYB113, and MYB114 (Figure 3B). In addition, both the amino acid signature [D/E]L × 2[R/K] × 3L × 6L × 3R, which is required for the interaction between the MYB and bHLH transcription factors [49], and the S amino acid residue critical for DNA binding of GL1 to GL2 [50], are full conserved in TmPAP1 and TmPAP2 (Figure 3B).

### 2.2. Expression of TmPAP2 in Arabidopsis Promotes Anthocyanin Biosynthesis

Considering that a dramatic difference in anthocyanin contents was observed in the “Buttercream” and the “Empress of India” nasturtium varieties (Figure 1), and that *TmPAP2* is the only regulator gene with a high expression level in the “Empress of India”, but undetectable in the Buttercream (Figure 3), it is very likely that TmPAP2 is a key regulator of anthocyanin biosynthesis.

**Figure 4:**
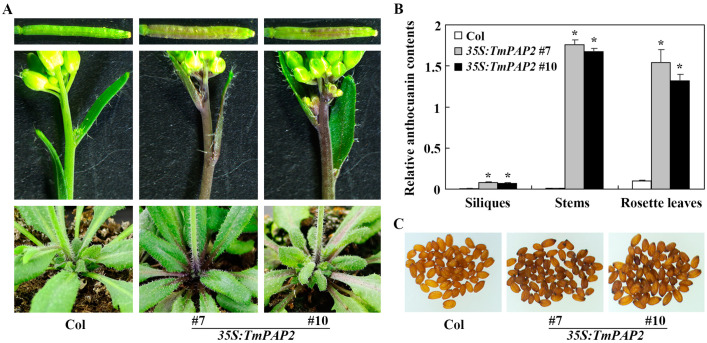
Anthocyanin accumulation in the Col wild-type and the *35S:TmPAP2* transgenic *Arabidopsis* plants. (**A**) Anthocyanin accumulation in the siliques (upper panels), stems (middle panels), and rosette leaves (lower panels) of 5-week-old soil-grown plants of the Col wild-type and the *35:TmPAP2* transgenic plants. (**B**) Relative anthocyanin contents in the Col wild-type and the *35S:TmPAP2* transgenic plants. Data represent the mean ± SD of three biological repeats. * Significant difference from the Col wild-type plants (*p* < 0.001). (**C**) Seed colors of the Col wild-type and the *35S:TmPAP2* transgenic plants. Pictures were taken under an OPTIKA microscope connected to a digital camera.

To examine if this is indeed the case, we generated transgenic *Arabidopsis* plants expressing *TmPAP2* under the control of the *35S* promoter. As shown in Figure 4A,B, an increased anthocyanin level was observed in the different organs of the transgenic plants, including the rosette leaves, stems, and siliques, as indicated by the color as well as the quantitative assays, indicating that the expression of *TmPAP2* promoted anthocyanin biosynthesis in *Arabidopsis*. In addition, the seeds of the transgenic plants showed a dark brown color, compared with the lighter brown color of the Col wild-type seeds (Figure 4C), suggesting that pro-anthocyanidin biosynthesis in *Arabidopsis* was also promoted by the expression of *TmPAP2*.

### 2.3. Expression Levels of the General Phenylpropanoid Pathway and Anthocyanin Biosynthesis Genes Are Increased in the TmPAP2 Transgenic Plants and the “Empress of India” Nasturtium

**Figure 5:**
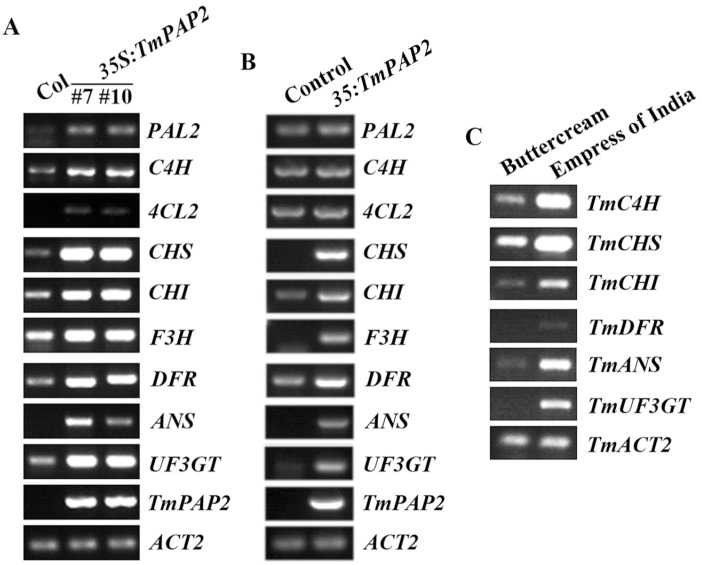
Expression of biosynthesis genes in the general phenylpropanoid pathway and anthocyanin biosynthesis pathway. (**A**) Expression of the biosynthesis genes in the Col wild-type and the *TmPAP2* transgenic *Arabidopsis* plants. RNA was isolated from 10-day-old seedlings and RT-PCR was used to examine the expression of the genes. The expression of *ACT2* was used as a control. (**B**) Expression of the biosynthesis genes in *TmPAP2* transfected protoplast. Protoplasts were isolated from the Col wild-type *Arabidopsis*, then the plasmid DNA of *TmPAP2* or *CAT* control was transfected into the protoplasts; the transfected protoplasts were incubated in darkness for 20–22 h, and then the RNA was isolated and used for RT-PCR, to examine the expression of the genes. The expression of *ACT2* was used as a control. (**C**) Expression of the biosynthesis genes in the “Buttercream” and the “Empress of India” nasturtiums. RNA was isolated from 7-day-old soil-grown seedlings, and RT-PCR was used to examine the expression of the genes. The expression of *TmACT2* was used as a control.

In *Arabidopsis,* the LBGs in the anthocyanin biosynthesis pathway are regulated by the MBW complex, formed by the R2R3 MYB transcription factor PAP1/PAP2/MYB113/MYB114, the bHLH transcription factor GL3/EGL3/TT8, and the WD40 protein, TTG1 [38,39,40,41,42,43,44]. Since the expression of *TmPAP2* promoted anthocyanin biosynthesis in *Arabidopsis* (Figure 4), we examined the expression LBGs in the transgenic plants. As expected, the expression levels of LBGs, including *DFR*, *ANS*, and *UF3GT,* were increased in the transgenic plants (Figure 5A). We also examined the expression of EBGs, including *CHS*, *CHI* and *F3H*, and found that their expression levels were increased in the transgenic plants (Figure 5A). Therefore, we further examined the expression of the enzyme genes in the general phenylpropanoid pathway and found that the expression levels of *PAL2*, *C4H* and *4CL2* were also increased in the transgenic plants (Figure 5A).

The expression of *TmPAP2* in *Arabidopsis* promoted the expression of enzyme genes, including LBGs and EBGs, in the anthocyanin biosynthesis pathway, as well as the enzyme genes in the general phenylpropanoid pathway, indicating that TmPAP2 may function alone to activate the expression of the genes. To examine if that is indeed the case, we examined the expression of these genes in transfected protoplasts, as we have previously shown that the MBW complex genes, including *TTG1*, *GL3,* and *EGL3*, are not expressed in protoplasts [51]. We found that even though only a slight if any increase was observed for enzyme genes in the general phenylpropanoid pathway, the expression levels of LBGs and EBGs in anthocyanin biosynthesis were greatly increased in protoplasts transfected with *35S:TmPAP2* plasmid DNA (Figure 5B). 

Having shown that TmPAP2 may function alone to regulate the anthocyanin biosynthesis genes in the transgenic *Arabidopsis* plant and transfected protoplasts (Figure 5A,B), we wanted to examine whether this is also the situation in nasturtium plants. Nasturtium homologs of *Arabidopsis*, C4H, CHS, CHI, DFR, ANS, and UF3GT, were identified and named TmC4H, TmCHS, TmCHI, TmDFR, TmANS, and TmUF3GT, respectively (File S1 in the Supplementary Materials). We found that relatively higher expression levels for all six genes were observed in the “Empress of India” nasturtium, and the expression of *TmC4H* and *TmDFR* was undetectable in the “Buttercream” nasturtium (Table S1 in the Supplementary Materials). RT-PCR results show that all six genes indeed showed higher expression levels in the “Empress of India” nasturtium compared to the “Buttercream” nasturtium (Figure 5C).

### 2.4. TmPAP2 Functions as a Transcription Activator

The expression levels of the general phenylpropanoid pathway and anthocyanin biosynthesis genes are higher in the “Empress of India” nasturtium (Figure 5C), while their expression levels increased in the *35S:TmPAP2* transgenic *Arabidopsis* plants, as well as in the transfected protoplasts (Figure 5A,B), suggesting that TmPAP2 may function alone as a transcription activator to regulate gene expression. Therefore, we examined the transcription activity of TmPAP2 in transfected protoplasts.

**Figure 6:**
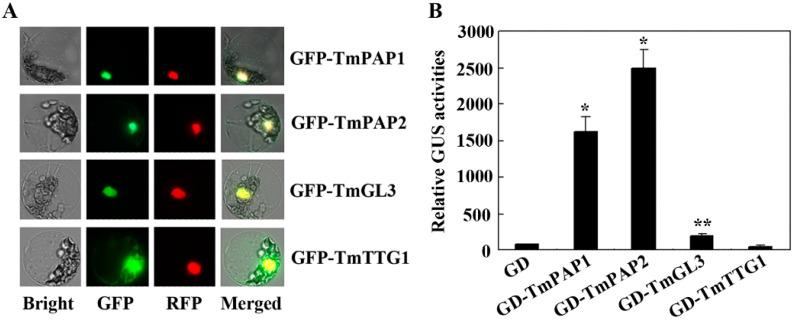
Subcellular localization and transcriptional activity of the nasturtium homologs of the *Arabidopsis* MBW complex proteins. (**A**) Subcellular localization of the nasturtium homologs of the *Arabidopsis* MBW complex proteins in *Arabidopsis mesophyll* protoplasts (with 400× magnificatio). Plasmids of constructs *GFP-TmPAP1*, *GFP-TmPAP2*, *GFP-TmGL3*, or *GFP-TmTTG1* were co-transfected with the nucleus indicator construct *NSL-RFP*, respectively, into protoplasts, and pictures were taken after the transfected protoplasts were incubated for 20–22 h in darkness. (**B**) Transcriptional activity of the nasturtium homologs of the *Arabidopsis* MBW complex proteins. Plasmids of the effector constructs *GD-TmPAP1*, *GD-TmPAP2*, *GD-TmGL3*, *GD-TmTTG1* or the control construct *GD* were co-transfected with the reporter construct *GAL4:GUS*, respectively, into the protoplasts, and GUS activity was assayed after the transfected protoplasts were incubated for 20–22 h in darkness. Data represent mean ± SD of three replicates. Significant differences from the GD control (* *p* < 0.001, ** *p* < 0.01).

The subcellular localization of TmPAP2 was examined first. Plasmids of the *GFP-TmPAP2* construct and the nucleus indicator construct *NLS-RFP* were co-transfected into *Arabidopsis* protoplasts, and the GFP fluorescence was examined. As shown in Figure 6A, GFP fluorescence was fully overlapped with RFP fluorescence, suggesting that TmPAP2 is a nucleus protein. We also examined the subcellular localization of TmPAP1, TmGL3, and TmTTG1, and found that they are all nucleus proteins (Figure 6A).

We then examined if TmPAP2 may activate reporter gene expression when recruited to the *Gal4* promoter region of the *Gal4:GUS* reporter gene by a fused *Gal4* DNA binding domain (GD). Plasmids of the effect construct *GD-TmPAP2* or the control construct *GD* were co-transfected, respectively, with the reporter construct *Gal4:GUS* into *Arabidopsis* protoplasts, and GUS activities were examined. As shown in Figure 6B, compared with the co-transfection of *GD*, GUS activity increased ~30-fold when *GD-PAP2* was co-transfected, suggesting that TmPAP2 functions as a strong transcription activator. Similarly, we found that GUS activity increased ~20-fold when *GD-PAP1* was co-transfected (Figure 6B), suggesting that TmPAP1 also functions as a strong transcription activator. On the other hand, GUS activity increased only ~2 folds when *GD-TmGL3* was co-transfected, whereas remained largely unchanged when *GD-TmTTG1* was co-transfected (Figure 6B), suggesting that TmGL3 may function as a weak transcription activator, but TmTTG1 does not confer any transcription activities.

### 2.5. TmPAP2 May Not be Able to Form an MBW Complex with TmTTG1 and TmGL3

The above results showed that TmPAP2 functions as a strong transcription activator (Figure 6B) and may function alone to regulate the expression of ABGs. However, since evidence from different plants suggests that anthocyanin biosynthesis is regulated by MBW complexes, we further examined if TmPAP2 may be able to form an MBW complex with TmGL3 and TmTTG1 by using protoplast transfection. 

**Figure 7:**
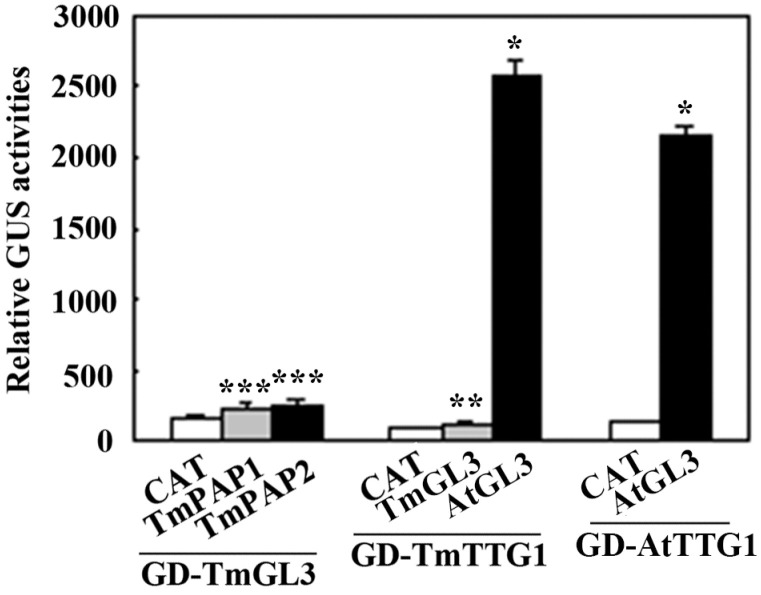
Interaction of the *Arabidopsis* MBW component proteins and their nasturtium homologs. Plasmids of the GD-fused constructs were co-transfected, respectively, with HA-fused constructs or control construct *CAT* and the *Gal4:GUS* reporter construct into protoplasts, and GUS activity was assayed after the transfected protoplasts were incubated for 20–22 h in darkness. Data represent mean ± SD of three replicates. Significant differences from GD control (* *p* < 0.0001, ** *p* < 0.01, *** *p* < 0.05).

Plasmids of the effector construct *GD-TmGL3* and reporter gene *Gal4:GUS* were co-transfected with the effector construct *TmPAP2* or with the control construct *CAT* into *Arabidopsis* protoplasts. In the system, TmGL3 is able to bind to the *Gal4:GUS* reporter gene via the fused GD domain, since TmPAP2 functions as a strong transcription activator; increased GUS activity will be observed if it can interact with TmGL3. However, only a slight increase in GUS activity was observed when *TmPAP2* was co-transfected (Figure 7). Similarly, a slight increase in GUS activity was observed when *TmPAP1* was co-transfected (Figure 7). We also found that a slight increase in GUS activity was observed when GD-TmTTGl was co-transfected with TmGL3, whereas GUS activity increased ~30-fold when *GD-TmTTG1* was co-transfected with *AtGL3* (Figure 7). As a control, GUS activity increased ~20-fold when the plasmids of the effector *GD-AtTTG1* and reporter *Gal4:GUS* were co-transfected with the effector construct *AtGL3* (Figure 7). These results suggest that TmPAP2 may not be able to form an MBW complex with TmTTG2 and TmGL3.

## 3. Discussion

The therapeutic and health benefits of nasturtiums are thought to be related to their high levels of secondary metabolites, including anthocyanins [1,9,11,16], an important group of water-soluble non-toxic natural pigments with antioxidant and anti-inflammatory properties [18,19,20,21,22]. We provided evidence in this study that the nasturtium R2R3 MYB transcription factor TmPAP2 is a key regulator of anthocyanin biosynthesis.

First, *TmPAP2* was highly expressed in the “Empress of India”, a high anthocyanin-containing nasturtium variety, but is undetectable in “Buttercream”, a low anthocyanin-containing nasturtium variety (Figure 1 and Figure 3). Second, the expression of *TmPAP2* in *Arabidopsis* promoted anthocyanin biosynthesis (Figure 4). Third, the expression levels of ABGs were increased in *Arabidopsis* transgenic plants expressing *TmPAP2* (Figure 5). Similarly, the expression levels of ABGs are higher in the “Empress of India” variety when compared with those in the “Buttercream” variety (Figure 5). Consistent with these observations, TmPAP2 functioned as a strong transcription activator (Figure 6). These results suggest that TmPAP2 is involved in the regulation of anthocyanin biosynthesis. It should be noted that a higher expression of *TmPAP1* was also observed in the “Empress of India” variety (Figure 3). TmPAP1 is closely related to TmPAP2, as well as PAP1, PAP2, MYB113, and MYB114 (Figure 2), the R2R3 MYB-type anthocyanin biosynthesis regulators in *Arabidopsis* [38], while TmPAP1 also functions as a strong transcription activator (Figure 6); therefore, it is very likely that TmPAP1 may also be involved in the regulation of anthocyanin biosynthesis. 

Furthermore, we found that TmPAP2 may function alone to regulate anthocyanin biosynthesis. TmPAP2 shared a highly conserved R2R3 domain with the *Arabidopsis* PAP, PAP2, MYB113, and MYB114, and it also contained a fully conserved [D/E]L × 2[R/K] × 3L × 6L × 3R (Figure 3), the amino acid signature required for the interaction between the MYB and bHLH transcription factors [49]. However, assays in the protoplasts show that TmPAP2 may not able to form an MBW complex with TmGL3 and TmTTG1 (Figure 7). It has been reported that R2R3 MYB transcription factors including MYB11, MYB12, or MYB111 alone are able to regulate the expression of EBGs in *Arabidopsis* [35,36], whereas the expression of LBGs is regulated by MBW complexes [37,38,39,40,41,42,43]. In addition, studies in recent years suggest that both LBGs and EBGs in several different plants are regulated by the MBW complexes [23,44,45,46]. We identified TmGL3 and TmTTG1, homologues of *Arabidopsis* GL3/EGL3 and TTG1, respectively (Figure 2). However, based on the results in transfected protoplasts, TmPAP2 may not able to interact with or may have only a very weak interaction with TmTmTTG1, whereas it is clearly that TmTTG1 is able to interact with *Arabidopsis* GL3 (Figure 7). In addition, TmPAP1 is also not able to interact with or has very weak interaction with TmGL3 (Figure 7). Consistent with this finding, the expression levels of ABGs, including EBGs and LBGs, were increased in the 35S:TmPAP2 plasmid DNA transfected *Arabidopsis* protoplasts (Figure 5). As the expression of the MBW genes TTG1, GL3, and EGL3 were not detectable in *Arabidopsis* protoplasts [51], these results suggest that TmPAP2 can function alone to regulate ABGs in the transfected protoplasts. However, we could not rule out the possibility that other R2R3 MYBs in nasturtiums may be able to interact with TmGL3, and other bHLH transcription factors may be able to interact with TmPAP2, thereby forming MBW complexes with TmTTG1 to regulate anthocyanin biosynthesis

However, considering that the expression levels of biosynthesis genes in the general phenylpropanoid pathway, including *PAL*, *C4H,* and *4CL,* were also increased in *35S:TmPAP2* transgenic *Arabidopsis* plants (Figure 5), and the expression level of *TmC4H* was much higher in the “Empress of India” variety than in the “Buttercream” variety (Figure 5), even though direct evidence is still required, it is very likely that TmPAP2 can function alone in nasturtiums to regulate anthocyanin biosynthesis via regulating the expression of biosynthesis genes in the general phenylpropanoid pathway and ABGs, including EBGs and LBGs. As a result, *TmPAP2* may be a good candidate gene for molecular breeding to increase the anthocyanin content in edible flowers, including nasturtiums, vegetables, and even crops, thereby increasing their therapeutic and health benefits.

## 4. Materials and Methods

### 4.1. Plant Materials and Growth Conditions

Seeds of available nasturtium varieties, including the “Empress of India” and “Buttercream” were obtained from Renee’s Garden (https://reneesgarden.com, accessed on 1 June 2020). To generate seedlings used for RNA isolation and anthocyanin content measurement, seeds of nasturtium plants were sown directly into soil pots and grown in a growth room with a 16 h light/8 h dark photoperiod at 22 °C and a photon density at ~120 μmol m^−2^ s^−1^.

The Columbia-0 (Col) *Arabidopsis* was used as the wild-type for plant transformation and protoplast isolation. The *35S:TmPAP2* transgenic plants were generated by transforming the Col wild-type *Arabidopsis* plants. For plant transformation, protoplast isolation, phenotype observation, and anthocyanin content measurement, seeds of the Col wild-type, and the *35S:TmPAP2* transgenic plants were sown directly into soil pots and grown in the growth room. For RNA isolation, seeds of the Col wild-type and the *35S:TmPAP2* transgenic plants were sterilized, plated on plates with 0.6% (*w*/*v*) phytoagar (PlantMedia, Dublin, OH), solidified 1/2 MS (Murashige & Skoog) medium with vitamins (Plant Media), and 1% (*w*/*v*) sucrose, kept at 4 °C in darkness for 2 days, and then transferred to the growth room.

### 4.2. RNA Isolation, Transcriptome Analysis, and RT-PCR

For comparative RNA-sequencing and RT-PCR analysis, RNA was isolated from 7-day-old soil-grown plants of the “Empress of India” and the “Buttercream” nasturtium varieties by using TRIzol^®^ reagent (Invitrogen, Carlsbad, CA, USA). RNA sequencing was performed by OriginGene (www.origin-gene.com, accessed 1 December 2020). To examine the expression of the general phenylpropanoid pathway biosynthesis genes and anthocyanin biosynthesis genes in *Arabidopsis*, RNA was isolated from 10-day-old seedlings of the Col wild-type and the *35S:TmPAP2* transgenic plants, and cDNA was synthesized as described previously, using the TransScript^®^ First-Strand cDNA Synthesis SuperMix kit (TransGene, Beijing, China) [52]. The primers used for examining the nasturtium genes are listed in Table S2 in the Supplementary Materials, and primers used for examining the *Arabidopsis* genes have been described previously [53].

### 4.3. Bioinformatics Analysis

Full-length amino acid sequences of the *Arabidopsis* anthocyanin biosynthesis regulators, including TTG1, GL3, EGL3, PAP1, PAP2, MYB113, and MYB114, and the general phenylpropanoid pathway and anthocyanin biosynthesis pathway biosynthesis enzymes, PAL, C4H, 4CL, CHS, CHI, F3H, DFR, ANS, and UF3GT were obtained from Phytozome (https://phytozome-next.jgi.doe.gov, accessed on 1 May 2021). The amino acid sequences were used to identify nasturtium homologs by using Local BLAST on BioEdit (unigene.pep) obtained by the RNA sequencing analysis; the corresponding CDS sequences were then obtained by checking the related gene ID in unigene.cds. The next most closely related proteins to TmPAP1, TmPAP2, and TmGL3 were also identified for phylogenetic analysis. The CDS and amino acid sequences for all the nasturtium homologs are listed in File S1 in the Supplementary Materials. The full-length amino acid sequences of related proteins were subjected to phylogenetic analysis on Phylogeny (http://www.phylogeny.fr/simple_phylogeny.cgi, accessed on 1 August 2022) by using the “One Click” mode with the default settings. The expression levels of the corresponding nasturtium homolog genes in the “Empress of India” and the “Buttercream” nasturtiums from RNA sequencing analysis are listed in Table S1 of the Supplementary Materials.

### 4.4. Constructs

The effect constructs *GD-AtTTG1* and *AtGL3*, the control effector constructs *GD* (*Gal4* DNA binding domain) and *CAT* (chloramphenicol acetyltransferase), the reporter construct *Gal4:GUS*, and the nucleus indicator construct *NLS-RFP* used for protoplast transfection were as described previously [54,55,56]. To generate the *GD-* and *GFP*-fused *TmAPA1*, *TmPAP2*, *TmGL3,* and *TmTTG1* constructs for protoplast transfection, the full-length ORF (open reading frame) sequences of *TmAPA1*, *TmPAP2*, *TmGL3,* and *TmTTG1* were amplified by RT-PCR using RNA isolated from the young leaves of 7-day-old soil-grown seedlings of the “Empress of India” nasturtium, digested with the proper enzymes and cloned in frames with a N-terminal GD or GFP tag into the *pUC19* vector, under the control of the CaMV *35S* promoter [57,58].

To generate the constructs for plant transformation, plasmids of the *GD-TmPAP2* construct in *pUC19* were digested with the proper enzymes and cloned into the *pUC19* vector with an N-terminal HA [55,56], then they were digested with the proper enzymes and cloned into the binary vector, *pTF101.1*.

### 4.5. Plant Transformation and Transgenic Plants Selection

The *35S:TmPAP2* transgenic plants were obtained by transforming the Col wild-type *Arabidopsis* plants with the *35S:TmPAPs* construct. The Col wild-type plants of ~5 weeks old, with a few mature flowers on the main inflorescence, were transformed via the *Agrobacterium tumefaciens* strain *GV3101* using the floral dip method [59]. 

T1 seeds collected from the transformed plants were sown directly into soil pots and the transgenic plants were selected when the seedlings were 10 days old by spraying with BASTA (0.01%, *w/v*). The T2 seeds collected from T1 transgenic plants and transgenic plant lines with 3:1 segregation were selected by spraying BASTA, and the homozygous plants were selected by spraying BASTA, from T3 seeds collected from the 3:1 segregation lines.

Multiple independent transgenic plants for the *35S:TmPAP2* construct were obtained, and phenotypes for the *35S:TmPAP2* transgenic plants were observed in the T1 generation and confirmed in the T2 and T3 generations. The expression of *TmPAP2* in the *Arabidopsis* plants was confirmed by RT-PCR, and two homozygous lines were used for the experiments.

### 4.6. Plasmid DNA Isolation, Protoplast Isolation, and Transfection

Protoplast isolation, plasmid DNA isolation, and protoplast transfection were performed as previously described [53,55,58]. In brief, protoplasts were isolated from rosette leaves collected from ~4-week-old Col wild-type *Arabidopsis* plants; plasmid DNA of the reporter and effectors constructs were isolated using an EndoFree Plasmid Maxi Kit (Omega Biotech, Shanghai, China) according to the manufacturer’s recommendations. The plasmid DNA that was thus isolated was transfected or co-transfected into the *Arabidopsis* protoplasts that were isolated. The transfected protoplasts were incubated in the dark and at room temperature for 20–22 h, then the GFP and RFP fluorescence were examined and pictures were taken under an FV1000 confocal microscope (Olympus, Monolith, Japan) with a magnification of 400×, The GUS activities were measured using a Synergy^TM^ HT fluorescence microplate reader (BioTEK, Winooski, VT, USA), and the expression of biosynthesis genes was examined by RT-PCR, as described previously [51].

### 4.7. Phenotype Observation

To examine anthocyanin biosynthesis in the *35S:TmPAP2* transgenic *Arabidopsis* plants, the transgenic plants were grown side by side with the Col wild-type plants in soil pots in a growth room, and photographs of the rosettes, inflorescence stems, and siliques were taken using a digital camera; the seed color was examined and photographs were taken under an OPTIKA microscope connected to a digital camera.

### 4.8. Anthocyanin Content Assays

To examine the anthocyanin content in the “Empress of India” and the “Buttercream” nasturtiums, anthocyanins were extracted from 6-day-old soil-grown nasturtium seedlings. To examine the anthocyanin content in the Col wild-type and the *35S:TmPAPs* transgenic plants, anthocyanins were extracted from the siliques, stems, and rosette leaves of adult soil-grown plants. Anthocyanin content was measured by following the procedure described previously [53].

### 4.9. Statistical Analysis

Statistical analysis was performed using Student’s *t*-test on GraphPad software (www.graphpad.com/quickcalcs/ttest1.cfm accessed on 29 August 2022).

## Data Availability

All data obtained were presented in this article and the supporting information.

## Supplementary Materials

The following are available online at https://www.mdpi.com/article/10.3390/ijms232012395/s1.
