# Supplementary material for: Tropaeolum majus R2R3 MYB Transcription Factor TmPAP2 Functions as a Positive Regulator of Anthocyanin Biosynthesis
Source: Int J Mol Sci. 2022 Oct 17;23(20):12395. doi: 10.3390/ijms232012395 (PMC9604057; doi:10.3390/ijms232012395)
Supplement: Supplementary file 1 [file ijms-23-12395-s001.zip › File S1.pdf]

Coding and amino acid sequences of the *Tropaeolum majus* genes identified in this study

TmPAP1
ATGGAAAACTACTCGGTAGGTTTGAGAAAAGGAGCATGGACTAGTGAGGAAGACGATCTATT
AAGGAAGTGCATACACAAATATGGAGAGGGAAAATGGCATCAAGTACCTCTTAAAGCAGGGT
TGAATAGATGCAGGAAAAGTTGTAGACTGAGGTGGTTGAATTATTTGAAACCAAACATCAAG
AGGGGAGAATTTGCTGAAGATGAGGTTGATTTGATTCTAAGGCTTCATAAGCTTCTCGGAAAC
AGGTGGTCATTAATCGCGGGTAGAATCCCGGGAAGAACGGCGAACGATATAAAAAATTACTG
GAACACCCGTCTCCGAGGAAAAGAATATCCTTTGAAAAAATATCCATACGTAACAAAAGGGA
AAACAATAGAGAAAAAAATAGAAGTAATTAAACCTCAACCTCGAGCCTTCACAAAAAATAAC
ATTTACTCCAAAAATCGTGTTGAGCATCCATTATGCATCAACGACAACCATTTGAAACCTCAA
CCGAAAACAAACCCAATGAACTCGATACCTAAGGACGAAAACGATGAGGACATTATGTGGTG
GAAAAGATTACTAGCCGAGTCCGAAACCGAAACCATATTGTTCAACGAAGAAACCCAGTCGG
GTGATCTTGGGTTTACGGGTGGAATCTTGGGTCATGATTTGGACCACATTTGGAGTTTTTTAAA
TGCAGATTCAGAAGATAATGTTGGTATTTGA

MENYSVGLRKGAWTSEEDDLLRKCIHKYGEGKWHQVPLKAGLNRCRKSCRLRWLNYLKPNIKR
GEFAEDEVDLILRLHKLLGNRWSLIAGRIPGRTANDIKNYWNTRLRGKEYPLKKYPYVTKGKTIEK
KIEVIKPQPRAFTKNNIYSKNRVEHPLCINDNHLKPQPKTNPMNSIPKDENDEDIMWWKRLLAESE
TETILFNEETQSGDLGFTGGILGHDLDHIWSFLNADSEDNVGI

TmPAP2
ATGGAAAGCTGCTTGGTAGGTTTGAGAAAAGGAGCATGGAGCAGTGATGAAGACGATCTATT
AAGGAAGTGCATACACAAATATGGAGAGGGAAAATGGCATCAAGTACCTCTTAAAGCAGGGT
TGAATAGATGCAGAAAAAGTTGTAGGCTGAGGTGGTTGAATTATTTAAAGCCAAACATCAAG
AGAGGAGAATTTGCAGAAGATGAGGTTGATTTGATTCTAAGGCTTCATAAGCTACTTGGAAAC
AGATGGTCTCTAATTGCTGGTAGAATCCCTGGAAGAACGGCGAATGATGTGAAAAACTATTG
GAACACTCGTGTACGCAAAACGAACCATCACTTGAGAAATTATCCGCACGAAACAAAAAGCA
TACATAAGGTGAAAGCAATCAAACCTCAACCGCAAGCTTTCAAAAATAATAACAAATACTCC
CAAGAAATACCTACAATCGCTAAAACACAAGAAAATGTTGTCGAAAAACGATTATGCATCGG
CGAAAACCATTTGAAACTTCCTACTACAACAACCACCATGATGTGTTCCATACCTACGGACGA
CAATGATGATGATATTACATGGTGGCAGAGATTATTAGCCGAGATCGAAACCGAAGAAGTAT
TGTTTGACAAAGAAATTCAGTCCGATGATGCGGGTTTCTCGAGTGGAGTTTTGGGTCATGATG
TGGACCAAGTTTGGAGTTTCTTAAATGATGATTTAGATTGA

MESCLVGLRKGAWSSDEDDLLRKCIHKYGEGKWHQVPLKAGLNRCRKSCRLRWLNYLKPNIKR
GEFAEDEVDLILRLHKLLGNRWSLIAGRIPGRTANDVKNYWNTRVRKTNHHLRNYPHETKSIHKV
KAIKPQPQAFKNNNKYSQEIPTIAKTQENVVEKRLCIGENHLKLPTTTTTMMCSIPTDDNDDDITW
WQRLLAEIETEEVLFDKEIQSDDAGFSSGVLGHDVDQVWSFLNDDLD

DN12074
ATGGAACGTTACGCAGGAGTGAGAAAAGGTAGATGGATCGAAGAAGAAGACAATCTTTTAAG
AAAATGTATTGAAAAGCATGGAGAAGGAAAATGGAGTGAAGTTCCTCTCAAGGCAGGCCTAA
ACAGATGCAGAAAAAGCTGTAGATTGAGGTGGTTGAACTATTTGAAGCCAGATATAAAAAGA
GGAAAATTCACTAAGGATGAATCTGATCTGATTATCAGACTTCATAAACTTTTGGGTAATAGA
TGGAAAATGATTGCTGATAGATTACCGGGAAGAACAGATAACGATATTAAGAATTATTGGAA
AGCTCATATCTTTAAGAAACTCGTCAATGAGAACAATGAAAAAGAAGAGATGATAAATGAAG
ATTTTTATCGCGACTATGTTGATAATGAATGTCAATACAAAAAAGATGAAGAATAA

MERYAGVRKGRWIEEEDNLLRKCIEKHGEGKWSEVPLKAGLNRCRKSCRLRWLNYLKPDIKRGK
FTKDESDLIIRLHKLLGNRWKMIADRLPGRTDNDIKNYWKAHIFKKLVNENNEKEEMINEDFYRD
YVDNECQYKKDEE

DN20611 (partical)
ATGGGGAGAGTTGCTTGGAGTGAAGAAGAAGATCTTTTGCTCAAGAAATACATAGAGCAATA
TGGAGAGGGGAAATGGCATCGTGTACCTCTTTTAACCGGACTTAATAGATGCCGGAAGAGCT
GTAGATTGAGATGGCTAAACTACTTACGACCCAATGTTAAAAGAGGGAGCTTTGCAGATGAT
GAAGCTGATATTATTATCAAGCTCCATGACATCTTGGGTAATAGGTGGTCACTAATAGCCGCA
AGACTACCGGGAAGAACGGCCAATGATGTCAAGAATTATTGGAATTGTCATTTGAGCAAAAG
ACTTAATATCAAAAATAATGAAAACACACCGACCAAAAAACCTCAACTTTCTAAAACAAGAA
CCAAGATCAACGTTAACATTACTGCAACAAGTTCAACAATTCCACAAGATCAAGAAACTAACT
CGTATTCAACACCAACGATGCACTACCTTTCACTTGATGGAGAGGAATGCAATGAGATACCAA
AA

MGRVAWSEEEDLLLKKYIEQYGEGKWHRVPLLTGLNRCRKSCRLRWLNYLRPNVKRGSFADDE
ADIIIKLHDILGNRWSLIAARLPGRTANDVKNYWNCHLSKRLNIKNNENTPTKKPQLSKTRTKINV
NITATSSTIPQDQETNSYSTPTMHYLSLDGEECNEIPK

TmGL3
ATGAGCAAGCTAATCGACGATTATCTGTTTGGTTCTTGTTTTATTGCTGAATCTTGTGGTGGGA
CTCATCTTCATCAGAGCGCATCCATTCAGACTGTTGTATGCTTTCCATTTTCGGGAGGTGTGGT
TGAGCTTGGCGTGACTGATATGGTGAAGGAGGATTTTTGTCTCATCACGTATGTCAAGACTTC
ATTCTTGGAAGTCCCCTACTCGATAGTCTCCGATATATTGGCTCCTCAGGTAGAGAAGACCCA
AACCGACCAAGTAATCCCTCATTCTGCGGTCGATCTTCAGATTCTTGATGATAATCTGATCCCA
GGCATCGGCTGCGAGGAGTTGCAAACGGTTTCTCATGATAGTAGCTCAAATGGCTTTGAAATG
GAACCAATGGAAGATTCACTCATACTCGAAGGGCTAAATGGTGAAGATTCTCGATTACAAAG
CTGGATATTCATGGAGGACGAGATCAGTAACTGTATCCATCAGAGTTCCAATGATGCCGATTG
TATATCTCAATCCTTTGTACAGCCTTTGGAGGTCATACCCGTTACACCGGTCGAAAAGGAAAA
CAGTCATTCCCTTCAAGACATTCAAGACAATAACCACACAAAACTGGCATCGGTTGATCTTCA
AGCCGATGACTTGCACTACCAAAGTGTTATCTTGACCCTTTTTAATAAGACTTCCCACCAGTTG
ATGTTGGGACCAAATGTGAAGAGATCTGACCACAAATCTAGCTTTGTTCCCTGGACGAAACGT
AGACGTGTGAACCCTCAGAAATCAGCTCACAAAACCCATCAAACACTACTGAAAAAAGTATT
ATTCAAAGTTCCTCGGACTTATGAAAACACAACGCTAGGTTTCCCAGATGGCACTGGGCTTAA
AGGTGTAGCTTGGAGGCCAAAAGTTGATGAAATCACAAACCATGTGTTATCGGAGAGGAGGC
GAAGAGATAATCTAAACCAAAGATTTACGGTTTTGAAATCTATAATCCCGACGATCACAAAG
GTTGACAAAGTATCTATTCTAGACGACACGATAGAATACCTCCAAGAACTTGAGAAAAGGGT
CGAAAAGTTGGAAGCTTGCAGAGAAACCGGTGAACTGGAAGCCGGAATGAGGAGAAAACAT
CACAATGCTGCAGAAAGAACATCTAATAACACAAACGATAAAACTACTAACAGAAAGAAATC
GTTGGTAAACAAGAGGAAAGCAAGTGACTACGATAACGCAGGTAAAGATGTTGACTTTGTTA
TACAGAAAGATGGTTCAGCCGATAACGTAAAGATTAGTACGAGTGACAACAATGTTGTGATT
GAGATCAAGTGTCCACAGAGAGAAGGAGTTATGCTCGATATCCTGGATGCAGCAAGCAGCCT
TCATTTAGATCCTTACTCAGTTCAATCATCTACTACTGATGAAATGATTTCTGTGACTATCCAA
TCTAAGTACAAAAGATCGAAATTGGCCTCAAGAGATCAGATCAAACAGGTACTTCAAAGAGT
TGCTTGGAATTCTTAA

MSKLIDDYLFGSCFIAESCGGTHLHQSASIQTVVCFPFSGGVVELGVTDMVKEDFCLITYVKTSFLE
VPYSIVSDILAPQVEKTQTDQVIPHSAVDLQILDDNLIPGIGCEELQTVSHDSSSNGFEMEPMEDSLI
LEGLNGEDSRLQSWIFMEDEISNCIHQSSNDADCISQSFVQPLEVIPVTPVEKENSHSLQDIQDNNHT
KLASVDLQADDLHYQSVILTLFNKTSHQLMLGPNVKRSDHKSSFVPWTKRRRVNPQKSAHKTHQ
TLLKKVLFKVPRTYENTTLGFPDGTGLKGVAWRPKVDEITNHVLSERRRRDNLNQRFTVLKSIIPTI
TKVDKVSILDDTIEYLQELEKRVEKLEACRETGELEAGMRRKHHNAAERTSNNTNDKTTNRKKSL
VNKRKASDYDNAGKDVDFVIQKDGSADNVKISTSDNNVVIEIKCPQREGVMLDILDAASSLHLDP
YSVQSSTTDEMISVTIQSKYKRSKLASRDQIKQVLQRVAWNS

DN6405
ATGATGGTCGATACACTGGTTTTAGACAACCTGTTTACAGATGACGTCGAGTTTAACCCAGAA
AGAACTAAAGCAAATGATGAATACAACATAGATTCCCCGACAGAATGTTCAAACGAGTGTGA

TCATAACCACCAGACAGAAGACTCATTTATGCTTGAAGGTATGAACGGTGGTATAGCTACTTC
TCAAGTCGAAAGCTGGAAATTCATAGCTGATGAATTCCCGGGTGGTGTTCGAAACTCTATAAC
CTCGAGTGATTGTATCTCTGAAGCTTATCTGAATCATGAAAACCGCGAAAAGGCCAGCAACAG
CCTTAATCACACAAAACTAAGCTCTTTACATCTTGGAGGGGGCGAAGAAGAAGAAGATTCAC
ACTACAGAAATACTCTTTCGGCTATTCTCAAGAGTCAAAGTTTGTTGACTAAAGCACGATGCT
TCGAAAGTTGTGATTTCAAGTCGAGCTTCGAGAGCTGGAAGAGAGGAGGAGGAGGAGGGGTT
TTTGATCGTCGTCGCTCGTGGTCGCGGACACCGTTGCCGCAGAAAGTTTTGAAAAAGGTGTTG
TTTGAAGTCCCTTTGATGTATGGTGGCTCTCCTCAGTCCCTAAAAGAAACTACCCAAAACGAC
TTTAACGGTATTAAGCGCGGCGGAGGACATGTTTCGGATAAACGAAAAGAAGACGAGAAGTT
TCTTGCACTACGATCGCTGGTTCCTTGCATTAGTGAGATTGACAAAGCAACAATATTAAGCGA
AACGATCAAGTACTTACGAGAGCTCGAAGCAAGAGTAGAGGAGTTAGAATCTTGCATGGATT
TCGAGGCGAGAACGAGAAGGAATCATGTAGACGTTATAGAGCGTACATCAGATAACTACGAG
TTTGATAAGGGGAAGAAGAGTTGGGTAAATAAGAGGAAGGCGCGCGATATTGATGAAGCTAG
ACCGGAATTTGATAGGATCGTCCATGAGGATGACATGATATCATCAAACAACTTGAAAGTTAT
TGTCAAAGAGCAAGAGGTTTCGATTGAGATGAGATGTCGTTATCGACCGTATATATTAGTCGA
TATCATGGATGCTATCAACAATCTACACTTGGATGCACACTCGGTTCGATCTTCTACACTTGAT
GGTTTTCTCACATTGACCCTCAACTCTAAGTTTCGAGGCACAGCAATAGCACCAGCTGGAATG
ATCAAGCGAGCTCTCCGGGAAGCAGCAGGGAGGTGCTGA

MMVDTLVLDNLFTDDVEFNPERTKANDEYNIDSPTECSNECDHNHQTEDSFMLEGMNGGIATSQ
VESWKFIADEFPGGVRNSITSSDCISEAYLNHENREKASNSLNHTKLSSLHLGGGEEEEDSHYRNTL
SAILKSQSLLTKARCFESCDFKSSFESWKRGGGGGVFDRRRSWSRTPLPQKVLKKVLFEVPLMYG
GSPQSLKETTQNDFNGIKRGGGHVSDKRKEDEKFLALRSLVPCISEIDKATILSETIKYLRELEARVE
ELESCMDFEARTRRNHVDVIERTSDNYEFDKGKKSWVNKRKARDIDEARPEFDRIVHEDDMISSN
NLKVIVKEQEVSIEMRCRYRPYILVDIMDAINNLHLDAHSVRSSTLDGFLTLTLNSKFRGTAIAPAG
MIKRALREAAGRC

TmTTG1
ATGGAGAATTCAACCCAAGAATCTCACCTAAGATCAGAGAATTCCATCACCTATGAATCCCCT
TACCCACTCTACGCCATGGCCGTATCTTCCTCTCCACCTCGTACCCAAAACCATTCTCAACGCA
TCGCCGTCGGTAGTTTCCTCGAGGACTACACAAACCGGGTTGATATAGTCAACTTCGATCTCG
ATTCGTTATCCATGAAGGCTCACCCGACTCTTTCATTCGACCATCCATACCCGCCTACAAAGCT
CATGTTTCACCCCAATTCACCATCAACCCTAAACTCATCTTTCGATCTCCTCGCCTCTTCCGGC
GATTTCCTCCGTCTCTGGGAGGTTCGAGATTCGTCAATCGAACCCGTAACGGTTCTCAACAAC
AGCAAGACAAGTGAATTCTGCGCTCCTCTCACTTCCTTCGATTGGAATGAGGTCGAGCCGAAG
AGGATTGGGACGTCGAGTATCGACACAACCTGCACGATTTGGGATATTGAAAGAGGTATCGTT
GAAACGCAGCTCATTGCGCACGACAAAGAGGTTTACGACATCGCTTGGGGAGAAGCTAGGGT
TTTCGCTTCGGTTTCCGCTGATGGATCGGTTCGAATTTTCGATTTAAGGGATAAAGAACACTCC
ACGATCATATACGAAAGCCCTCAACCTGATACCCCTCTGCTTAGATTAGCTTGGAACAAACAG
GATTTGAGGTACATGGCGACGATTTTGATGGATAGTAATAAGGTTGTGATTTTGGATATACGA
TCTCCATCAATGCCGGTTGCTGAGTTGGAGAGGCATACCTCGAGCGTTAACGCTATAGCTTGG
GCTCCACAGTGTCCTCGGCACATTTGCTCCGCCGGAGATGACTCGCAAGCGCTTATTTGGGAG
GCTGCCCCAACGGTGGCTGGACCTAACGGAATTGACCCAAAAGGCCATGTACTCGGCTGGTCC
GAGATTAACCAGCTCCAATGGTCCTCCGCTCAGCCTGATTGGATTGCTGTTGCCTTCTCCAACA
AGATGCAGCTATTGAAAGTTTAA

MENSTQESHLRSENSITYESPYPLYAMAVSSSPPRTQNHSQRIAVGSFLEDYTNRVDIVNFDLDSLS
MKAHPTLSFDHPYPPTKLMFHPNSPSTLNSSFDLLASSGDFLRLWEVRDSSIEPVTVLNNSKTSEFC
APLTSFDWNEVEPKRIGTSSIDTTCTIWDIERGIVETQLIAHDKEVYDIAWGEARVFASVSADGSVRI
FDLRDKEHSTIIYESPQPDTPLLRLAWNKQDLRYMATILMDSNKVVILDIRSPSMPVAELERHTSSV
NAIAWAPQCPRHICSAGDDSQALIWEAAPTVAGPNGIDPKGHVLGWSEINQLQWSSAQPDWIAVA
FSNKMQLLKV

TmLWD1
ATGGGCGTGAGCAGCGACCCAAATCCAGACGGATCCGACGAGCAGCAGAAACGGTCGGAGA
TCTACACTTACGAGGCACCATGGCATATATACGCTATGAACTGGAGTGTTCGTCGAGACAAGA

AGTACCGTCTCGCAATAGCCAGCCTCGTCGAGCAGTACCCTAACCGTGTTGAAATAGTCCAGC
TTGACGATTCTAATGGCGAGATTCGATCCGACTCTAGTCTCTCTTTCGAACACCCTTATCCCCC
ACAAACCATATTCATACCCGACAAAGACTGCCAAAAACCAGATCTACTTGCTACTTCCGGTGA
TTTCTTACGGATATGGCGCATCAACGACGACGACGACGATGACAACTTCCAGTCCAAGGGTTC
TAAGGTTGAGCTCAAGAGTGTTCTTAATGGTAACAAGAACAGCGAGTTTTGCGGGCCACTCAC
TTCCTTTGACTGGAACGAAGCTGAGCCAAGGCGTATTGGTTCCTCTAGCATCGACACCACATG
TACGATATGGGATATCGAGAGGGAAACGGTTGATACCCAGCTTATTGCCCATGACAAGGAAG
TATACGACATAGCTTGGGGCGGCGTTGGTGTCTTCGCTTCTGTCTCAGCAGATGGTTCCGTTAG
GGTTTTTGATCTCCGAGACAAGGAGCACTCAACTATAATATACGAGAGCTCCGAACCTGACAG
TCCTTTAGTTCGTCTCGGATGGAACAAGCAAGATCCAAGGTACATGGCTACTATAATTATGGA
CAGTGCCAAGGTTGTAGTCCTGGATATTCGCTTCCCTACACTTCCCGTAGTTGAGCTCCAGAG
ACACCAAGCTAGCGTCAACGCTATTGCATGGGCCCCACACAGCTCCTGCCACATATGCACGGC
CGGTGATGATTCCCAAGCCTTGATTTGGGACCTCTCCTCCATGGGTCAGCCCGTTGAAGGAGG
GCTTGATCCCATTTTGGCTTACACTGCTGGAGCAGAAATCGAACAACTGCAGTGGTCTTCTTCC
CAACCGGATTGGGTAGCCATCGCATTCTCCACTAAGCTTCAGATTCTGAGGGTATGA

MGVSSDPNPDGSDEQQKRSEIYTYEAPWHIYAMNWSVRRDKKYRLAIASLVEQYPNRVEIVQLD
DSNGEIRSDSSLSFEHPYPPQTIFIPDKDCQKPDLLATSGDFLRIWRINDDDDDNFQSKGSKVELKS
VLNGNKNSEFCGPLTSFDWNEAEPRRIGSSSIDTTCTIWDIERETVDTQLIAHDKEVYDIAWGGVG
VFASVSADGSVRVFDLRDKEHSTIIYESSEPDSPLVRLGWNKQDPRYMATIIMDSAKVVVLDIRFPT
LPVVELQRHQASVNAIAWAPHSSCHICTAGDDSQALIWDLSSMGQPVEGGLDPILAYTAGAEIEQL
QWSSSQPDWVAIAFSTKLQILRV

TmC4H
ATGGATCTCCTCCTCTTGGAGAAGGCGCTGTTAGCGACATTCATTGCAGCGGTAATCGCCATT
ACCATATCGAAGCTCCGTGGAAAGCGGTTCAAGCTTCCTCCGGGTCCAATACCGGTACCTGTG
TTCGGTAACTGGCTTCAAGTTGGCGATGATCTTAACCACCGGAATCTTTCCGAAATGGTGAAG
AAATACGGACAGATTTTCCTTCTGCGGATGGGCCAACGGAACCTCGTGGTGGTTTCATCGCCG
GAGCTTTCGAAGGAAGTGTTGCACACGCAGGGGGTTGAGTTCGGGTCGAGGACTCGAAATGT
TGTGTTTGATATTTTCACAGGTAAGGGGCAGGATATGGTGTTCACGGTGTACGGTGAGCACTG
GAGGAAGATGAGGAGGATCATGACCGTTCCTTTCTTCACTAACAAGGTTGTTCAACAGTATCG
TTACGGGTGGGAAGATGAGGCACGTCACGTGGTTGAGGATGTGAAGAAGAATCCAGAAGCGG
CTACAAATGGAATCGTGCTGAGGAAACGGTTACAATTGATGATGTATAACAATATGTACAGG
ATTATGTTTGATAGAAGATTCGAGAGTGAGGATGATCCTCTGTTCCTTCAGCTCAAAGCGGTG
AATGGAGAAAGGAGTAGATTGGCTCAGAGCTTCGAGTATAACTACGGCGATTTCATTCCAATC
TTGAGGCCGTTTTTGAGAGGTTACTTGAAGATCTGTAAGGAGGTTAAGGAGAGACGATTGAA
GCTCTTCAAGGATTACTTCATTGACGAGAGGAAGAAACTTGCTAGCACTACTGGCACGAACAA
CGAAGGATTGAAATGCGCCATTGATCATATGATCGAAGCTCAAGAAAAGGGGGAGATCAATG
AAGATAACGTTCTTTACATTGTTGAGAATATCAACGTTGCTGCTATCGAAACAACTTTGTGGTC
AATTGAATGGGGCATTGCCGAGCTTGTGAACAACCCTGATGTCCAGAGAAAACTCCGAGCTG
AACTCGACAAAGTTCTAGGACCCAATCAAATCACCGAGCCCGACATTGAAAAACTCCCTTACC
TTCAGGCTGTCGTGAAAGAAACTCTCAGGTTTCGAATGGCGATTCCGCTCCTCGTCCCTCACAT
GAACCTCCACGATGCTAAGCTTGGCGGCTTTGACATCCCCGCAGAGAGCAAAATCTTGGTGAA
TGCATGGTGGCTAGCAAACAACCCCGACAACTGGAAAAAACCAGAAGAATTCAGGCCGGAGA
GGTTCTTGGAAGAGGAATCAAAAGTTGAAGCTAATGGAAACGACTTCCGTTTCATTCCGTTTG
GTGTCGGAAGGAGGAGTTGCCCGGGAATCATTCTTGCGTTGCCTATTCTTGGTATCACTTTGG
GGCGGTTGGTGCAGAACTTTGAGCTGTTGCCTCCTCCGGGACAGGCGAAAATCGATACTTCTG
AGAAAGGTGGGCAATTCAGCTTGCACATTTTGAAGCACTCTACCATTGTTTTGAAGCCAAGGT
CATATGTTTAA

MDLLLLEKALLATFIAAVIAITISKLRGKRFKLPPGPIPVPVFGNWLQVGDDLNHRNLSEMVKKYG
QIFLLRMGQRNLVVVSSPELSKEVLHTQGVEFGSRTRNVVFDIFTGKGQDMVFTVYGEHWRKMR
RIMTVPFFTNKVVQQYRYGWEDEARHVVEDVKKNPEAATNGIVLRKRLQLMMYNNMYRIMFDR
RFESEDDPLFLQLKAVNGERSRLAQSFEYNYGDFIPILRPFLRGYLKICKEVKERRLKLFKDYFIDER
KKLASTTGTNNEGLKCAIDHMIEAQEKGEINEDNVLYIVENINVAAIETTLWSIEWGIAELVNNPD
VQRKLRAELDKVLGPNQITEPDIEKLPYLQAVVKETLRFRMAIPLLVPHMNLHDAKLGGFDIPAES

KILVNAWWLANNPDNWKKPEEFRPERFLEEESKVEANGNDFRFIPFGVGRRSCPGIILALPILGITL
GRLVQNFELLPPPGQAKIDTSEKGGQFSLHILKHSTIVLKPRSYV

TmCHS
ATGTCTGGTTTAGCAGCATCATCACCATCCATTGAAGAGATCAGAAAATCCCAAAGAGCTGAT
GGTCCGGCCAACATCTTAGCCATCGGTACAGCCACCCCTAACCACTGCATCACTCAGTCTGAG
TATCCTGATTACTACTTCAGGATCACCAACAGTGAACACATGACTGATCTCAAGGAGAAGTTC
AAACGCATGTGTGATAAATCGCAGATAAAGAAAAGGTATATGCATTTGACGGAAGATTTCTT
GAAGCAGAATCCAAACATGTGTGCTTACATGGCGCCATCTCTAGATGCACGTCAAGATATTGT
TGTGTTGGAGGTGCCGAAGCTTGGGAAAGAAGCTGCGGTGAAAGCTATTAAGGAATGGGGAC
AGCCGAAATCGAAGATCACGCATTTGGTTTTCTGCACTACTTCCGGCGTCGACATGCCCGGAG
CCGATTACCAACTTCTTAAGCTGCTAGGTCTTCGTCCGTCCGTCAAACGTTTGATGATGTATCA
ACAAGGCTGTTTCGCCGGCGGTACCGTCCTTCGTTTGGCGAAGGATCTCGCCGAGAATAATAA
AGGTGCACGTGTCCTCGTCGTTTGCTCCGAGATCACTGCTGTCACTTTCCGAGGACCGTCGGA
GACTCATCTGGACTCGCTTGTCGGTCAAGCGCTTTTCGGTGACGGAGCGGCGGCGCTCATTGT
CGGTGCTGATCCGGACGTATCGATCGAGCGACCTTTGTTTCAGGTTGTGTCGGCTGGTCAGAC
GATCTTACCGGACTCAGACGGCGCGATCGACGGACACTTGCGTGAAGTTGGTTTAACGTTTCA
TCTACTGAAAGATGTACCGACATTGATCTCTAAGAACATTGAGAAGAATTTGGTCGAGGCGTT
TGGTCCACTCGGGATCAATGATTATAACGGGGTGTTCTGGATCGCGCATCCGGGTGGACCGGC
GATTTTGGATCAAGTTGAGTTGAAGTTGGGTTTGGAAAAGGAGAAGTTGAGGTCGACTCGAG
AGGTGTTGAGCGAGTACGGAAACATGTCGAGTGCTTGCGTTTTGTTCATATTGGATGAAATGA
GGAAGAAATCGGTTGAAGAAGGGAAAACAACAACCGGTGAAGGGTTGGATTGGGGTGTTCTG
TTTGGGTTTGGACCCGGTTTAACGGTTGAAACGGTTGTGTTACACAGTGTTCCGATTCAACCA
ACTATTTGA

MSGLAASSPSIEEIRKSQRADGPANILAIGTATPNHCITQSEYPDYYFRITNSEHMTDLKEKFKRMC
DKSQIKKRYMHLTEDFLKQNPNMCAYMAPSLDARQDIVVLEVPKLGKEAAVKAIKEWGQPKSKI
THLVFCTTSGVDMPGADYQLLKLLGLRPSVKRLMMYQQGCFAGGTVLRLAKDLAENNKGARVL
VVCSEITAVTFRGPSETHLDSLVGQALFGDGAAALIVGADPDVSIERPLFQVVSAGQTILPDSDGAI
DGHLREVGLTFHLLKDVPTLISKNIEKNLVEAFGPLGINDYNGVFWIAHPGGPAILDQVELKLGLE
KEKLRSTREVLSEYGNMSSACVLFILDEMRKKSVEEGKTTTGEGLDWGVLFGFGPGLTVETVVLH
SVPIQPTI*

TmCHI
ATGGCAACGTCACCGTCACCGTCGCCGTCATCGGTCGTCACCGGAGTCCAAATCGAGAACATT
GCGTTCCCGCCTACTGTTAAGCCTCCGGGATCCACAAAAACTCTGTTTCTAGCTGGCGCAGGG
GCGAGAGGGTTGGAGATCCAAGGCAATTTCGTGAAGTTTACAGCGATAGGGGTTTACTTAGA
AGATACAGCTCTCTCGGCTATGGCTGCGAAGTGGAAAGGGAAAACTGCTGAGGAATTGACGG
AATCCGTTGAGTTTTTCCGGGACATTGTCACAGGTCCCTTCGAGAAATTCACACAGGTGACAA
TGATCTTGCCACTAACCGGTGCTCAATACTCAGAGAAGGTAAGCGAAAACTGTGTTGCAATCT
GGAAGTTGCTCAGTATTTACACTGATGCAGAAGCCAAAGCCATTGAAAAATTTCTAGAGGTTT
TGAAGGATGAAACATTCCCACCGGGCACATCTATTCTCTTCACCCAAACACCAAATGGATCAT
TAACAATTAGCTTTTGGAAAGATGGATCGATACCAGAAGTGGCGAATGCAGTGATAGATAAC
AAACTACTTGCAGGAGCAATACTGGAGTCTATTATTGGTAAGCATGGTGTTTCTCCTGAAGCA
AAGCAGAGCTTGGCTTTGAGATTTTGGGAGTTGTTAAAGGAGTTTGAATCCGATGAAGGTAAA
CCAGAAGTTAAGAAGGTGACAGGAGAAGAGGTCGATGCCGGAAAAGTAACTGCT**TGA**

MATSPSPSPSSVVTGVQIENIAFPPTVKPPGSTKTLFLAGAGARGLEIQGNFVKFTAIGVYLEDTALS
AMAAKWKGKTAEELTESVEFFRDIVTGPFEKFTQVTMILPLTGAQYSEKVSENCVAIWKLLSIYTD
AEAKAIEKFLEVLKDETFPPGTSILFTQTPNGSLTISFWKDGSIPEVANAVIDNKLLAGAILESIIGKH
GVSPEAKQSLALRFWELLKEFESDEGKPEVKKVTGEEVDAGKVTA*

TmDFR
ATGGATTTCGAGTCTAAAGATCCTGAGAACGAAGTGATAAAGCCAACAATAAATGGGGTGTT
AAACATAATGAAAGCATGTGCAAAAGCAAAAGGTGTGAAGAGGCTTGTGTTCACTTCATCAG
CCGGGACTGTGGGCGTCGAACAAAATCAGAAACTGGTTTATGATGAGACTAATTGGAGTGAC

TTGGAATTTATCCAAACCAAAAAGATGACTGGATGGATGTACTTTGTGTCCAAAACTTTGGCA
GAACAAGCAGCCTGGAAGTTTGCTAAGGATAACAACATTGATTTAATCAGTATCATACCATCT
CTTGTTGTGGGTCCATTTATCATGCAAACTATGCCACCAAGTCTTATCACTGCACTTTCCCTTA
TTACAAGAAACGAACCACATTATTCAATCATAAAACAATGTCAATATGTTCATCTTGATGACC
TATGTAATGCTCATATATATTTGTTCGAGAATCCTAAAGCTGAAGGTCGATACATTTGCTCGTC
AAACCATTGTACCATCTTTGATATTGTCAAATTGCTCAACAAGAAGTATCCTGAATACAATGT
ACCTACAAAGTTTAAAGACATGGACGATAGTTTGAAGAGTATTGCTTTCAGCTCGAAAAAGAT
TATCGAATTAGGGTTCGAGTTCAAATATAGTTTGGAGAGCATGTTTGTAGGAGCTGTTGAGAC
ATGTCGCGTGAAAGAATTGATACCGCTTTCTTATAAGGACGAACAACCAAATAACAACGTCA
AAGCTGAATTTCTTACGCAAGTCAATGAGAAATTTGAGGAGATCAATGTTTGA

MDFESKDPENEVIKPTINGVLNIMKACAKAKGVKRLVFTSSAGTVGVEQNQKLVYDETNWSDLEF
IQTKKMTGWMYFVSKTLAEQAAWKFAKDNNIDLISIIPSLVVGPFIMQTMPPSLITALSLITRNEPH
YSIIKQCQYVHLDDLCNAHIYLFENPKAEGRYICSSNHCTIFDIVKLLNKKYPEYNVPTKFKDMDDS
LKSIAFSSKKIIELGFEFKYSLESMFVGAVETCRVKELIPLSYKDEQPNNNVKAEFLTQVNEKFEEIN
V

TmANS
ATGGCTATGCTTGGTGGTTCTCTTGCTGTTCCAAATGTTCAAGAAATGGTTAAACAACCAATC
ACTACTATTCCTTCCAGATTTCACTACCATGATCATCCAGACCCTTCTTTGATTATATCCAAAC
CAACTTCTTCACCACAAGTTCCAATCATTGATATGCACAAACTGTTATCAGGAGACTCCATGG
AAACTGAACTCCATAAGTTGCATCTTGCTTGTCAAGAGTGGGGCTTCTTTCAGTTGGTGAATC
ATGGAGTAAGCATAGAGTTGATGGAGAAAGTGAAATCAGAAACTGAAAAATTCTTCAATTTA
CCAATTGAAAAGAAGAAAGAATATTGGCAACAACCAGGAGTCGATATGGAGGGGTTCGGACA
AGCTTTTGTAAAATCGGAACAACAAAAACTAAACTGGTTAGATATGTTCTACATCGTTACTCA
ACCTAAATCTTTGAGAAAACCTCGTCTTTTCCCGAAGCTCCCTACTTCTTACAGAGATACCATG
GATGCTTATTCGGATGAAGTTAAAAATCTAGCATGGGAAATACTTAAGTTGATGGCAAAAGC
ATTAAATATGGAGGTGAAGGATTTAAATGGATTATTTGGAGATATGGCTCAAGCCATGACAAC
AAACTACTACCCACCATGTCCAGAACCAGACCGTGTGATTGGACTGAAACCACACTCTGACGC
TGGTGGTCTAACAATCCTTCTCCAAGTTGGTGACATGGAAGGACTCCAACTCAAGAAAGATGG
AATTTGGATTCCTGTTAGACCTCTTCCAAATGCTTTTATACTCAACATTGGAGACTGTTTGGAG
ATCGTCACGAATGGAATTTACCATAGCAATGTGCATCGTGCTGTTGTGAACTCGGTTAAAGAA
AGGATATCTATTGCTACATTTTGTAACCCCAAACTAGATGAAGTTATGGGTCCGGCACCAAGC
TTGATTACCCCTCAAACTCCTGCAGTATTTAAGGAAATCTGTGTCGCGGAATACTTAAAGGGG
TTTTTTTCGGTTGACTTGAAAGGGAGATCATATATTGATGTTGTGAGGGTTAATCAAAACGAA
AATGCATAA

MAMLGGSLAVPNVQEMVKQPITTIPSRFHYHDHPDPSLIISKPTSSPQVPIIDMHKLLSGDSMETEL
HKLHLACQEWGFFQLVNHGVSIELMEKVKSETEKFFNLPIEKKKEYWQQPGVDMEGFGQAFVKS
EQQKLNWLDMFYIVTQPKSLRKPRLFPKLPTSYRDTMDAYSDEVKNLAWEILKLMAKALNMEVK
DLNGLFGDMAQAMTTNYYPPCPEPDRVIGLKPHSDAGGLTILLQVGDMEGLQLKKDGIWIPVRPL
PNAFILNIGDCLEIVTNGIYHSNVHRAVVNSVKERISIATFCNPKLDEVMGPAPSLITPQTPAVFKEIC
VAEYLKGFFSVDLKGRSYIDVVRVNQNENA

TmUF3GT
ATGGATTTCCTTCCTGGGTTTTCCGCCATCCGTACCATGGACATGCCCGAAGGCGTAATAACC
GAAGACCTTAAATCTCCGATTCCATCAATGCTAAACAAGCTCGGAACATATCTTCCACGCGCA
GCAGCAGTTCCCACAAACTCCTATGAAGAACTAGATCCACCTGTAGTCCAAATCCTCAAATCA
AGATTTCGCTTCATCAACATCGGTCCCTTCGCGTTAACGTCGTCGTCATCGCCGCCGTCATCCA
ACTCAGACGACCAACACGGATGTCTAACATGGCTAGACACCCAAGCGCCAGCATCCGTCGCC
TACATCAGTTTCGGCAGCGTGATAACGCCGCCACCGCAAGAGCTCGCCGCGTTAGCCGAAGC
GTTAGAGGAAAGGAACATCCCGTTTCTTTGGTCGTTTCGAGGGAATCCCGAGAAACATTTCTC
CGAAGAGTTTTTGTCCGCCGGAAAGTTTGTACCGTGGGCTCCGCAGGTGAAAGTATTGGAACA
TAAATCCGTCGGCGTTTTCGTCACGCATTGCGGATGGAACTCTGTGTTGGAAAGTATCATCGG
TGGCGTTCCGATGATTTGTAGGCCTTTTTTTGGGGATCAACCGCTCAATGTACGTACGGTAGA
GGCTGATTGGGGAGTCGGTGTGGGGATCGAAGGTGGTAGATTTACGAAAGATGGAACAAAAA

AAGCTTTGGAATCGGTGATTTCGAGCGATCGAGGGAAGATAATGAGAAAGAAACTTGTAGCG
TATAGAGAACAAGCTTTGAATGCTGTAAAACCAGGAGGTAGTTCGGCTGAAAATTTTAAGATC
TTGGTAGATATTGTGACAAAGTAA

MDFLPGFSAIRTMDMPEGVITEDLKSPIPSMLNKLGTYLPRAAAVPTNSYEELDPPVVQILKSRFRFI
NIGPFALTSSSSPPSSNSDDQHGCLTWLDTQAPASVAYISFGSVITPPPQELAALAEALEERNIPFLW
SFRGNPEKHFSEEFLSAGKFVPWAPQVKVLEHKSVGVFVTHCGWNSVLESIIGGVPMICRPFFGDQ
PLNVRTVEADWGVGVGIEGGRFTKDGTKKALESVISSDRGKIMRKKLVAYREQALNAVKPGGSS
AENFKILVDIVTK

TmACT2
CDS 1134bp
ATGGCAGAAGGTGAGGATATCCAGCCACTCGTCTGTGACAATGGAACAGGAATGGTCAAGGC
TGGATTCGCTGGTGACGATGCTCCCAGGGCTGTGTTTCCTAGCATTGTAGGTCGCCCACGTCA
CACTGGTGTAATGGTTGGGATGGGCCAAAAGGATGCATATGTCGGTGATGAGGCTCAATCCA
AGAGGGGTATTTTAACACTGAAATACCCAATTGAGCATGGTATTGTGAACAACTGGGATGATA
TGGAGAAGATTTGGCATCACACCTTCTATAATGAACTTCGAGTGGCTCCAGAAGAACATCCAG
TTCTTCTTACTGAAGCACCTCTGAATCCAAAGGCCAATCGTGAAAAAATGACCCAAATCATGT
TTGAGACCTTTAATACTCCTGCTATGTATGTCGCCATCCAGGCTGTTCTCTCCCTTTATGCCAG
TGGTCGTACAACTGGTATTGTTCTGGACTCTGGTGATGGTGTCAGCCACACTGTCCCTATCTAT
GAGGGATACGCTCTCCCACACGCCATCCTCCGTCTCGATCTTGCAGGTCGTGACCTTACCGATT
GCCTCATGAAAATCTTAACAGAGCGTGGCTATTCCTTCACCACTACAGCAGAGCGTGAAATCG
TGAGGGACATGAAGGAGAAGCTGGCATACATAGCTCTCGACTATGAGCAGGAGCTGGAAACC
TCCAAGACTAGTTCTTCTGTTGAGAAGAGCTATGAGTTGCCTGATGGACAGGTGATCACCATT
GGTGCTGAGCGTTTCAGATGCCCAGAGGTTCTCTACCAGCCATCCATGATCGGAATGGAAGCT
GCGGGTATTCATGAGACCACATACAACTCTATCATGAAGTGTGATGTCGATATCAGGAAGGAC
TTATACGGAAACATTGTCCTTAGTGGTGGGTCAACCATGTTCCCTGGTATCGCTGACAGAATG
AGCAAGGAAATTACTGCTCTCGCCCCAAGCAGCATGAAAATTAAGGTGGTAGCTCCACCCGA
GAGGAAGTACAGTGTCTGGATCGGTGGCTCTATCTTAGCATCTCTCAGCACTTTCCAGCAGAT
GTGGATTGCAAAGGCTGAATACGATGAATCAGGTCCATCTATTGTGCACAGGAAATGCTTCTA
A

TmACT2-F: GTTGGGATGGGCCAAAAGGA
TmACT2-R: CGACCACTGGCATAAAGGGA
