# Supplementary material for: Tropaeolum majus R2R3 MYB Transcription Factor TmPAP2 Functions as a Positive Regulator of Anthocyanin Biosynthesis
Source: Int J Mol Sci. 2022 Oct 17;23(20):12395. doi: 10.3390/ijms232012395 (PMC9604057; doi:10.3390/ijms232012395)
Supplement: Supplementary file 1 [file ijms-23-12395-s001.zip › Table S2.pdf]

Table S2 Nasturtium gene primers used in this study

Primer Name	Sequence
TmPAP1-Nde1F	5'- CAACATATGGAAAACTACTCGGTAGG -3'
TmPAP1-Sac1R	5'- CAAGAGCTCAAATACCAACATTATCTTC -3'
TmPAP2-Nde1F	5'- CAACATATGGAAAGCTGCTTGGTAGG -3'
TmPAP2-Sac1R	5'- CAAGAGCTCAATCTAAATCATCATTTAAG -3'
TmGL3-Nde1F	5'- CAACATATGAGCAAGCTAATCGACGA -3'
TmGL3-Sac1R	5'- CAAGAGCTCAAGAATTCCAAGCAACTCTTTG -3'
TmTTG1-Nde1F	5'- CAACATATGGAGAATTCAACCCAAGAATC -3'
TmTTG1-Sac1R	5'- CAAGAGCTCAAACTTTCAATAGCTGCATCTTG -3'
TmACT2-F	5'- GTTGGGATGGGCCAAAAGGA -3'
TmACT2-R	5'- CGACCACTGGCATAAAGGGA -3'
TmC4H-RTF	5'- ACTACTGGCACGAACAACGAA -3'
TmC4H-RTR	5'- ATACCAAGAATAGGCAACGCA -3'
TmCHS-RTF	5'- GTGTCCTCGTCGTTTGCTCC -3'
TmCHS-RTR	5'- CACCCCAATCCAACCCTTCA -3'
TmCHI-NdeIF	5'- CAACATATGGCAACGTCACCGTCACCGTC -3'
TmCHI-SacIR	5'- CAAGAGCTCAAGCAGTTACTTTTCCG -3'
TmDFR-NdeIF	5'- CAACATATGGATTTCGAGTCTAAAGATC -3'
TmDFR-SacIR	5'- CAAGAGCTCAAACATTGATCTCCTC -3'
TmANS-RTF	5'- CCAACTTCTTCACCACAAG -3'
TmANS-RTR	5'- CTCCAAACAGTCTCCAATG -3'
TmUF3GT-RTF	5'- ATCTCATTACCTTCATCCCCA -3'
TmUF3GT-RTR	5'- AAAACTGACCTTCAATCTCCC -3'
